# Observational Study of Clinical Profiles and Management of Liver Abscess in Hospitalized Patients: A North Indian Tertiary Care Perspective

**DOI:** 10.7759/cureus.54401

**Published:** 2024-02-18

**Authors:** Shishirendu S Parihar, Aakash S Shah, Nitesh Bassi, Ishan Mittal, Dawesh Yadav, Vinod K Dixit, Anurag K Tiwari

**Affiliations:** 1 Gastroenterology, Institute of Medical Sciences, Banaras Hindu University, Varanasi, IND

**Keywords:** spontaneous liver abscesses, tailored treatment, amoebiasis, intra-abdominal infections, liver abscesses

## Abstract

Background

Liver abscesses are a significant health concern, necessitating prompt diagnosis and appropriate management. Spontaneous liver abscesses are a frequent reason for hospitalizations in India, particularly in the northern part. By analyzing demographics, symptoms, radiological findings, laboratory parameters, and treatment outcomes, this study will contribute valuable insights to enhance the understanding and management of liver abscesses.

Aims and objective

To evaluate demographic, clinical, laboratory, and radiological parameters and management options in hospitalized patients with liver abscesses at a tertiary care center.

Methods

This study retrospectively analyzed prospectively collected data from 150 patients diagnosed with liver abscesses who were admitted to our ward for one year. Data on demographic characteristics, clinical presentation, etiology, radiological findings, laboratory investigations, management strategies, and treatment outcomes were collected. Descriptive statistics and relevant statistical tests were employed for data analysis.

Results

The study population had a mean age of 40.28±12.72 years, with a male preponderance (136 (90.7%)). Amoebic abscesses (94 (62.7%)) were the most common. Hepatomegaly (144 (96%)), fever (140 (93.3%)), abdominal pain (136 (90.7%)), and anorexia (118 (78.7%)) were the most common symptoms. Ultrasonography revealed solitary abscesses (99 (66%)) to be more common than multiple abscesses (24 (16%)), with a predominant location in the right lobe (128 (85.3%)). Laboratory investigations showed leukocytosis in 121 (80.7%), elevated liver enzymes (95 (63.3%) aspartate aminotransferase (AST) and 80 (53.3%) alanine transaminase (ALT)), elevated alkaline phosphatase (ALP) in 133 (88.7%), and low albumin levels (138 (92%)) in a significant proportion of patients. Single-time needle aspiration (95 (63.3%)), percutaneous drain (36 (24%)), and surgical intervention (4 (2.7%)) were the primary treatment modalities. Serum albumin level (p<0.001) and ALP (p<0.001) were significantly low and high, respectively, in patients with hospital stays ≥10 days.

Conclusions

This study provides insights into patients with liver abscesses' clinical and laboratory parameters and management strategies. The findings highlight the diverse clinical presentation, varied etiologies, and the importance of radiological imaging and laboratory investigations in diagnosis and management. Tailored treatment strategies based on the patient's condition are crucial for optimizing outcomes.

## Introduction

Liver abscesses are serious and potentially life-threatening infections characterized by localized collections of pus within the liver parenchyma [1]. Prompt diagnosis and appropriate management are crucial for optimizing patient outcomes [2]. Not all hepatic abscesses require hospitalization; however, management in an outpatient setting with antibiotics always carries a risk of complications, leading to delayed recovery and increasing morbidity, mortality, and cost of treatment. A greater understanding of the patient’s profile is needed to address the knowledge gaps in tertiary care centers.

Liver abscesses can be broadly classified as amoebic liver abscess (ALA) and pyogenic liver abscess (PLA). These abscesses may arise spontaneously or secondary to some intrabdominal infections (e.g. cholangitis, appendicitis, etc.), trauma, and invasive interventions involving the hepato-biliary system [1]. Sometimes, cystic masses of the liver, like hydatid cysts, may become secondarily infected by bacteria and present like PLA. ALA typically arises in the setting of amoebic colitis, leading to the migration of parasites through the portal circulation to the liver. However, patients with ALA do not frequently present with colitis features. Escherichia coli, Klebsiella pneumonia, and Enterococcus species were the most common isolates from PLA aspirates. Accurate diagnosis relies on imaging techniques and laboratory investigations in the background of clinical features and predisposing conditions [1]. Management typically involves antimicrobial therapy, percutaneous drainage (needle aspiration or catheter drainage), and rarely, surgical intervention [3]. However, the need for such interventions and their timing requires further exploration.

In the present study, we included 150 patients with liver abscesses (both PLA and ALA) hospitalized over one year at a tertiary care center to describe clinical, laboratory, and radiological parameters and to evaluate management options in hospitalized patients with liver abscesses. This study will contribute to valuable insights into the understanding and management of liver abscesses in tertiary care settings, ultimately improving patient care and outcomes.

STROBE (Strengthening the Reporting of Observational Studies in Epidemiology) guidelines were adhered to during the preparation of this manuscript.

## Materials and methods

Patients

This retrospective observational study was conducted at the Department of Gastroenterology, Banaras Hindu University, a leading tertiary-care center with expertise in liver diseases. The present study included 150 consecutive patients diagnosed with liver abscesses based on radiological and biochemical analysis between May 2022 to April 2023. Patients with a liver abscess secondary to trauma or surgical intervention and due to cholangitis were excluded. Being a retrospective, observational study, ethical approval was not obtained.

Detailed data from a prospectively stored patient’s record were collected on (A) demography (age, gender, relevant comorbidities, and socioeconomic status), (B) clinical presentations reported at the time of admission, including fever, abdominal pain, anorexia, hepatomegaly, splenomegaly, ascites, pleural effusion, jaundice, and pallor, (C) imaging (abscess volume and location and the number of abscesses), (D) laboratory investigations (complete blood count, liver function tests and kidney function test), (E) management strategies (details of the treatment interventions employed, such as needle aspiration, percutaneous drainage procedures, or surgical interventions) in addition to medical treatment, and (F) complications (pleural effusions, rupture, organ failures, vascular complications, etc).

Hospitalization and treatment protocol

There is no consensus guideline on when to admit a patient with a liver abscess. We generally admit those who do not respond clinically to oral antibiotics therapy in 48 to 72 hours, require drainage of the abscess, have organ failure, have complicated abscesses, such as rupture to the pleural or peritoneal cavity, and have vascular complications and sepsis. Indications of drainage that we followed were non-response to antibiotic therapy (oral or injectable) for 48-72 hours, left lobe abscess, large abscesses > 5 cm size on imaging, complicated abscess, and superficial abscess impending rupture. The method of drainage was either single or multiple needle aspiration or percutaneous catheter drainage (PCD). Rupture to the pleural or peritoneal cavity was managed by pleural or peritoneal drains. Aspirated pus was subjected to microscopy and culture. In case of deterioration, despite these measures, surgical consultations were taken, and patients were managed accordingly by the team. In addition to supportive care, antibiotics were given to all patients. We follow a regimen of metronidazole 750 mg thrice a day (oral/injectable) for 10-14 days plus amoxicillin-clavulanic acid thrice daily (dosing as per route) for 14 days for ALA. For PLA, a broad-spectrum antibiotic (meropenem or a third-generation cephalosporin) along with anaerobic coverage (usually metronidazole) were given initially. Changes in antibiotics were done as per culture and sensitivity reports or physician’s preferences based on clinical assessment.

Statistical analysis

All the data were analyzed using IBM SPSS ver. 25 (IBM Corp., Armonk, NY, USA). Descriptive statistics were used to analyze the collected data. Continuous variables were reported as mean ± standard deviation. Categorical variables were summarized as frequencies and percentages. Means were compared using one-way analysis of variation (ANOVA), whereas for categorical variables, the chi-square test or Fisher's exact test was applied. A P value < 0.05 was considered statistically significant.

## Results

Demographic and clinical profile

Table 1 describes and compares clinical characteristics based on the nature of the abscess. A total of 150 patients with liver abscesses were included in this study and analyzed. The mean age of patients was 40.28±12.72 years, with male preponderance (136 (90.7%)). The majority of the patients belonged to the lower socio-economic class (96 (64%)), followed by the middle class (51 (34%)), and 3 (2%) patients were from the upper-middle/upper class. Alcohol use was present in 80 (53.3%) patients, and 19 (12.7%) patients were diabetic.

**Table 1:** Comparing clinical characteristics of liver abscess based on the nature of the abscess All the data are expressed as mean ± standard deviation, * data are expressed as frequency. One-way analysis of variance (ANOVA) was used to obtain statistical significance. A P value of <0.05 is considered significant.

Characteristics	Amoebic	Pyogenic	Tubercular	P value
Age (years)	41.39±12.27	48.80±13.90	36.0±4.38	<0.001
Sex (male/female)*	102/8	28/6	6/0	0.045
Abscess volume (mL)	418.65±248.30	270.50±115.68	595.0±432.70	0.001
Duration of hospitalization (days)	11.95±5.76	11.15±4.74	9.5±1.64	0.358
Hemoglobin (g/dl)	9.29±2.42	10.33±1.04	8.99±2.19	0.033
Total leukocyte counts (/microliters)	16489.47±7222.60	15197.5±5913.94	21930.0±1452.12	0.081
INR	1.43±0.24	1.28±0.24	1.43±0.14	0.120
Bilirubin	1.88±1.99	1.15±0.90	0.75±0.05	0.063
Aspartate aminotransferase (IU/L)	83.87±102.04	78.05±69.64	63.5±3.83	0.914
Alanine transaminase (IU/L)	63.0±67.84	70.15±66.56	26.5±2.82	0.413
Alkaline phosphatase (IU/mL)	668.59±356.89	479.55±277.85	534.5±319.32	0.036
Serum albumin (g/dL)	2.60±0.55	2.63±0.44	2.25±0.38	0.349
Blood urea (mmol/L)	35.71±25.22	23.75±9.16	18.5±2.7	0.079
Serum creatinine (mg/dL)	0.925±0.23	0.815±0.16	0.70±0.10	0.015

**Figure 1:**
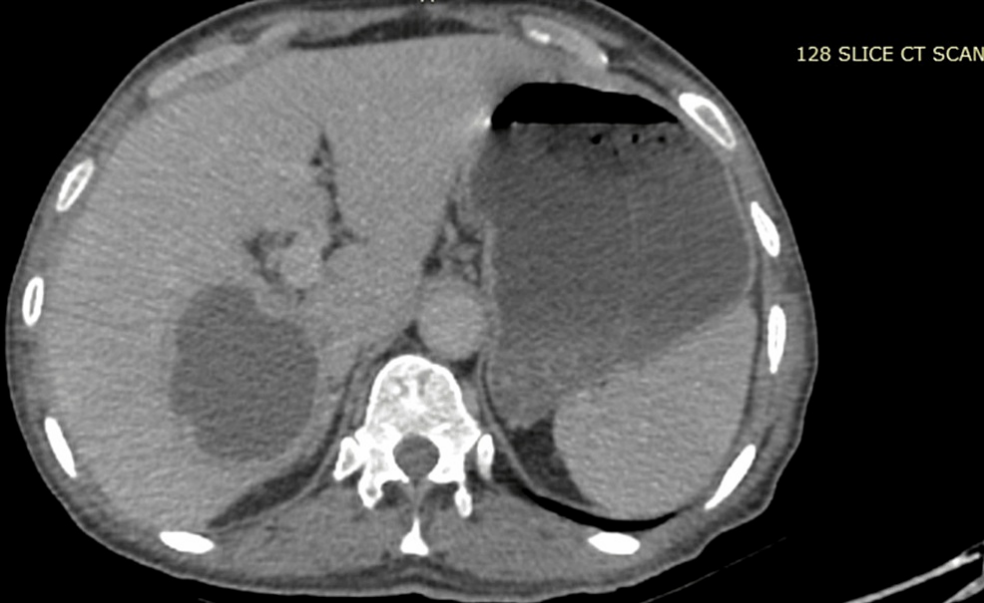
Axial section of contrast-enhanced CT Shows a well-defined hypodense lesion in segment 7 of the right lobe of the liver, with an internal non-enhancing area (suggestive of necrosis)

**Figure 2:**
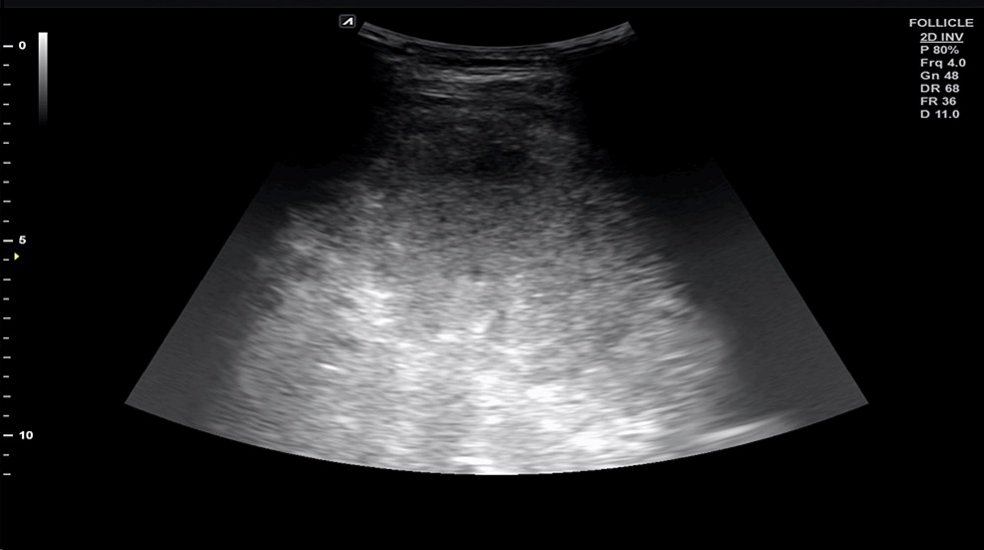
Ultrasound sonographic picture of liver section Shows an ill-defined heterogeneous hypoechoic collection in the left lobe of the liver, with free-floating echoes suggestive of internal necrosis

Table 2 provides the clinical profile of all patients. Fever (140 (93.3%)) and abdominal pain (136 (90.7%)) were the most common presenting symptoms, and hepatomegaly and anorexia were present in 144 (96%) and 118 (78.7%) patients, respectively. Lower gastrointestinal bleeding was present in only 11 (7.3 %) of patients. The majority reported amoebic abscess (94 (62.7%)), followed by pyogenic (41 (27.3%)) and tubercular (6 (4%)). Mixed abscess was reported in 9 (6%) patients. Ultrasonography revealed solitary abscesses (99 (66%)) to be more common than multiple abscesses (24 (16%)). Figure 1 shows the axial section of contrast-enhanced CT, which shows a well-defined hypodense lesion in segment 7 of the right lobe of the liver, showing an internal non-enhancing area (suggestive of necrosis). Figure 2 is the ultrasound sonographic picture of the liver section, which shows an ill-defined heterogeneous hypoechoic collection in the left lobe of the liver, showing free-floating echoes suggestive of internal necrosis.

**Table 2:** Clinical profile of all patients with a liver abscess NA: data not available

Symptoms and signs	Frequency	Percentage	Mean duration
Fever	140	93.3	21.69±16.62 days
Abdominal pain	136	90.7	19.97±16.89 days
Anorexia	118	78.7	NA
Nausea/vomiting	99	66	NA
Cough	55	36.7	NA
Breathlessness	34	22.7	NA
Abdominal distension	21	14	NA
Bleeding per rectum	11	7.3	NA
Diarrhea	8	5.3	NA
Hepatomegaly	144	96	NA
Pallor	89	59.3	NA
Icterus	43	28.7	NA
Weight loss	43	28.7	NA
Splenomegaly	20	13.3	NA

Involvement of the right lobe was predominant (128 (85.3%)) followed by both the right and left lobes (13 (8.7%)) and the left lobe alone (9 (6%)). The mean duration of hospitalization was 13.47±6.93 days.

**Figure 3:**
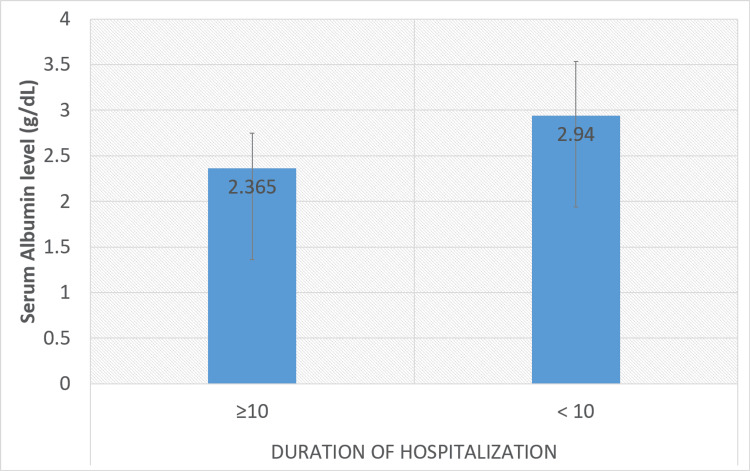
Association between duration of hospitalization (days) and serum albumin level P value <0.001. The normal albumin range is 3.4 to 5.4 g/dL. P value was calculated using an independent sample t-test.

**Figure 4:**
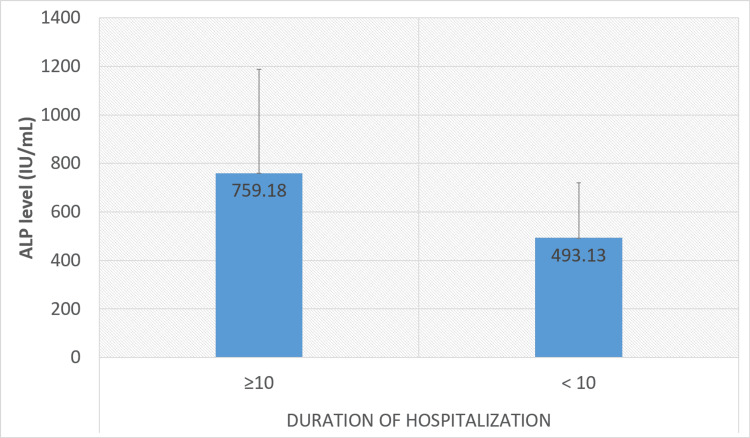
Association between duration of hospitalization (days) and ALP level. P value =<0.001. Normal ALP range is 35-130 IU/mL. P value was calculated using the independent sample t-test. ALP: alkaline phosphatase

Table 3 provides the details of the laboratory investigations performed on all the patients. Laboratory investigations showed leukocytosis in 121 (80.7%), elevated liver enzymes (95 (63.3%) AST and 80 (53.3%) ALT), elevated ALP in 133 (88.7%), and low albumin levels (138 (92%)) in a significant proportion of patients. Single-time needle aspiration (95 (63.3%)), percutaneous drain (36 (24%), and surgical intervention (4 (2.7%)) were the primary treatment modalities. Serum albumin level (p<0.001) and ALP (p<0.001) were significantly low and high, respectively, in patients with hospital stays ≥10 days. Figure 3 and Figure 4 show the association between the duration of hospitalization (days) and serum albumin and ALP levels, respectively.

**Table 3:** Laboratory investigations in patients with a liver abscess, highlighting out-of-range percentages Hb: hemoglobin, TLC: total leukocyte count, AST: aspartate aminotransferase, ALT: alanine transaminase, ALP: alkaline phosphatase, INR: international normalized ratio Out-of-range cut-off means a deranged/abnormal level of the parameter.

Parameters	Minimum	Maximum	Mean	SD	Reference range	Out-of-range cut-off	Out-of-range percentage
Hb (g/dL)	3.80	13.20	9.5871	2.07420	Male: 13.8 to 17.2 (g/dL) Female: 12.1 to 15.1 g/dL	<11 gm/dL	68.0
TLC (/mm)	5810	46700	18533.27	8641.054	5000-10,000/mm	>11000/μL	80.7
INR	0.90	2.59	1.5032	.33765	1.1 or below	>1.2	76.7
Bilirubin (mg/dL)	0.4	18.0	2.793	3.7634	< 0.3 mg/dL	>1.2 mg/dL	44.0
AST (U/L)	20	729	129.14	149.956	Men: 14 to 20 units/L. Women: 10 to 36 units/L.	>50 IU/L	63.3
ALT (U/L)	14	443	94.87	98.142	4 to 36 U/L	>50 IU/L	53.3
ALP (IU/L)	171	2056	732.30	435.310	4 to 147 IU/L	300 IU/L	88.7
Serum albumin (g/dl)	1.74	3.90	2.5184	.53269	6 to 8 g/dl	<3.5 g/dL	92.0
Blood urea (mg/dL)	14	205	57.43	53.496	5 to 20 mg/dL	<40 mg/dL	56.7
Serum creatinine (mg/dL)	0.5	8.8	1.303	1.2161	0.7 to 1.3 mg/dL	<1.5 mg/dL	75.3

Table 4 provides the details of the general study outcome in details. Single-time needle aspiration was performed in 95 (63.3%), percutaneous drain in 36 (24%), and both interventions were performed in 15 (10%) patients. Surgical intervention was done in four (2.7%) patients.

**Table 4:** General study outcome reported in the study population PCD: percutaneous catheter drainage

General study outcome	Frequency	Percentage
Received intervention	120	80
Resolved with antibiotics	30	20
Received only PCD	54	36
Received only aspiration	12	8
Received aspiration followed by PCD	51	34
Received surgical intervention	3	2

**Figure 5:**
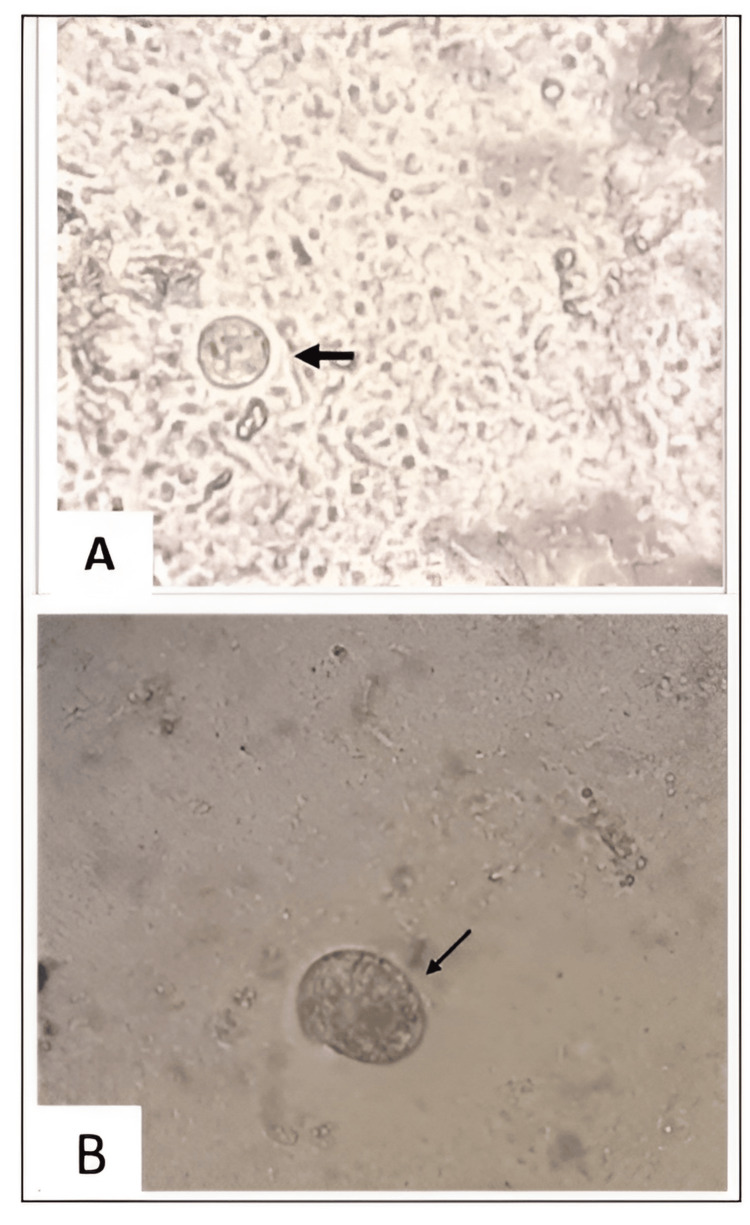
Microscopic image (40x) showing (A) Entamoeba cyst and (B) trophozoite Image taken from Entamoeba: Species, Classification and Biology. Nova Science Publishers, Inc. 978-1-53618-506-5, 1-51. Permission and license obtained from Nova Science Publishers, Inc.

The most common isolates were Enterobacter species (8/41), Streptococcus pyogenes (8/41), and Staphylococcus aureus (10/41). Other aerobic isolates were Escherichia coli (6/41). Proteus mirabilis (7/41) and Pseudomonas aeruginosa (2/41) were the anaerobic isolates found in our study. Figure 5 shows an example microscopic image (40x) of an Entamoeba cyst (Figure 5A) and trophozoite (Figure 5B).

Enterobacter species were sensitive to sulphamethoxazole+ trimethoprim, piperacillin/tazobactam, and amoxicillin, Staphylococcus aureus to moxifloxacin, amikacin, ciprofloxacin and sulphamethoxazole + Trimethoprim, Escherichia coli to piperacillin/tazobactam, ceftazidime, meropenem and colistin, Pseudomonas aeruginosa to ceftazidime, Streptococcus pyogenes to cefixime, cefuroxime, and doxycycline, and Proteus mirabilis to gentamicin, piperacillin/tazobactam, and ceftazidime.

**Figure 6:**
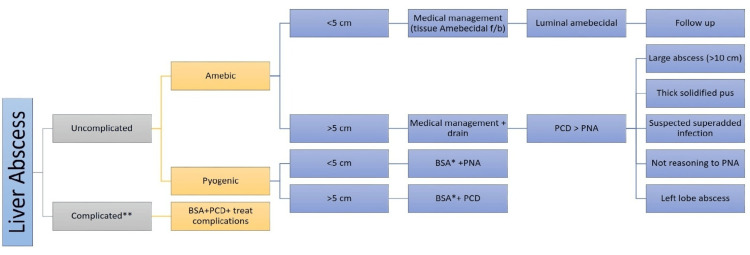
Algorithm for the management of patients with a liver abscess * Antibiotic to be modified according to culture sensitivity; ** Ruptured or impending rupture, <5 cm from the liver margin. BSA: broad-spectrum antibiotic; PNA: percutaneous needle aspiration; PCD: percutaneous drainage Image credits: Dr. Shishirendu S Parihar

Table 5 provides details on the complications of liver abscesses in the study population. Mortality was reported in four (2.7%). Those who died had low serum albumin (n=2), increased ALP (n=2), mixed liver abscesses (n=3), old age (n=4), and other comorbidities (n=3), jaundice (n=2), INR (n=1), encephalopathy (n=1), and intraperitoneal rupture (n=1). Figure 6 describes the proposed algorithm for the management of patients with liver abscesses.

**Table 5:** Local and systemic complications of liver abscesses in the study population HV: hepatic vein, IVC: inferior vena cava

Complications			Frequency	Percentage
Local	Pleural Effusion		90	60
	Abscess Rupture	Biliary rupture	6	4
		Intraperitoneal rupture	11	7.3
		Intrapleural rupture	23	15.3
	Venous thrombosis	HV thrombosis	6	4
		Portal venous thrombosis	6	4
		IVC thrombosis	1	0.7
Systemic complication		Pneumonia	13	8.7
		Urosepsis	7	4.7
		Encephalopathy	1	0.5
		Acute kidney injury	17	11.3

## Discussion

The demographic characteristics of the study population revealed male preponderance, with a mean age of 40.28±12.72 years. These findings are consistent with Indian studies such as by Sharma et al. (40.5 years) and Mukhopadhyay et al. (43.64 years) with predominant male involvement, and highlight the need to consider age and gender in the assessment and management of liver abscesses [4,5]. High alcohol intake by young men may predispose them to LA, explaining the gender and age discrepancies. Alcohol inhibits liver Kupffer cells, which remove amoeba. Invasive amoebiasis also requires free iron [6]. A diet strong in carbohydrates and iron, often from country spirits in chronic drinkers, predisposes to invasive amoebiasis [7]

Amoebic abscess (58.7%) was the most common, followed by pyrogenic in the present study. An Indian study from BHU reported a higher prevalence of amoebic liver abscesses in their study population, highlighting low socio-economic status and the habit of alcohol consumption as important predictors [8].

The clinical presentation of liver abscesses is diverse and often nonspecific. In our study, fever (93.3%) was the predominant symptom, followed by abdominal pain (90.7%), hepatomegaly (96%), and anorexia (78.7%). Various studies quote abdominal pain and fever in 62-94% and 67-87%, respectively [4,5]. These findings align with the typical clinical features of liver abscesses [2]. However, it is important to note that the clinical presentation can vary, and some patients may present with atypical symptoms or be asymptomatic, leading to diagnostic challenges.

Abdominal ultrasound plays a crucial role in diagnosing and characterizing liver abscesses. Its sensitivity to detect the LA ranges from 92% to 97% [6,9].

Our study revealed that right-lobe solitary abscesses were more common than multiple abscesses. Similar patterns of involvement were reported by Sharma et al. [4] and Mukhopadhyay et al. [5]. This highlights the importance of abdominal ultrasound in guiding accurate diagnosis and targeted interventions. Portal circulation causes LA to prefer the right lobe. Most blood drains from the right colon, where intestinal amoebiasis occurs. This region has many colonic problems that predispose to LA. Right lobe blood flow volume and biliary canaliculi are higher, causing congestion [5,10].

Laboratory investigations provide valuable information in the evaluation of liver abscesses. Aggressive LA patients' mainly alcoholic etiology have significant leukocytosis, hyperbilirubinemia, hypoalbuminemia, increased liver enzymes, and elevated alkaline phosphatase [11]. In our study, leukocytosis and elevated liver enzymes were commonly observed, reflecting the inflammatory response and liver involvement. A significant proportion of patients reported high ALP and low albumin levels, which are poor prognostic markers in patients with LA. Accordingly, earlier investigations found high alkaline phosphatase levels in 70-80% of cases, regardless of illness severity or duration [12,13]. In the present study, serum albumin levels (p<0.001) and ALP (p<0.001) were significantly low and high, respectively, in patients with hospital stays≥10 days. Similarly, Jindal et al. reported longer hospital stays in patients with deranged laboratory markers [14]. Previous studies also advocated the importance of early aspiration to reduce the hospital stay. A previous meta-analysis by Chavez-Tapia et al. of seven RCTs reported a shorter duration of hospitalization in aspirated patients [15].

Complications

Several complications may arise in the setting of liver abscess, including sepsis and organ failures, leading to prolonged hospitalizations and mortality. In our study, the most common complication was pleural effusion (60 %), which may, in this setting, be reactionary or secondary to rupture of the abscess in the pleural cavity (15%).

Managing liver abscesses requires a multidisciplinary approach, and our study demonstrated that single-time needle aspiration was the primary treatment modality, followed by percutaneous drainage and surgical intervention (as some were complicated by rupture). Inaccessible anatomical location, inability to respond to conservative therapy, and complications, including peritonitis, biliary-enteric fistulization, and others, might require surgery. A combination of single-time needle aspiration and drainage was utilized in some cases. Single-time needle aspiration and hospital stay duration varied based on individual patient responses and complications. These findings emphasize the need for tailored management approaches based on the severity and characteristics of each case [4].

Limitations

Despite the valuable insights gained from this study, a few limitations should be acknowledged. The retrospective design introduces inherent limitations and potential biases associated with data collection. This study describes data from a single center without any control group, and hence, comparative evaluation could not be done. These factors may limit the generalizability of the findings to other settings. Therefore, caution should be exercised in extrapolating these findings to different patient populations and healthcare settings.

## Conclusions

This study comprehensively evaluates the clinical, laboratory, and management profiles of patients with liver abscesses. The findings underscore the importance of considering demographic characteristics, clinical presentation, etiology, radiological findings, and laboratory investigations in assessing and managing liver abscesses. Further prospective studies involving larger cohorts from multiple centers are warranted to validate these findings and guide evidence-based approaches to optimize patient outcomes.
