# Correction: Observational Study of Clinical Profiles and Management of Liver Abscess in Hospitalized Patients: A North Indian Tertiary Care Perspective

**DOI:** 10.7759/cureus.c167

**Published:** 2024-03-22

**Authors:** Shishirendu S Parihar, Aakash S Shah, Nitesh Bassi, Ishan Mittal, Dawesh Yadav, Vinod K Dixit, Anurag K Tiwari

**Affiliations:** 1 Gastroenterology, Institute of Medical Sciences, Banaras Hindu University, Varanasi, IND

This article has been corrected to include proper attribution and permission information for Figure 5.

